# Label-Free Quantitative Proteomics Reveal the Mechanisms of Young Wheat (*Triticum aestivum* L.) Ears’ Response to Spring Freezing

**DOI:** 10.3390/ijms242115892

**Published:** 2023-11-02

**Authors:** Weiling Wang, Yuting Zhang, Chang Liu, Yongwen Dong, Xue Jiang, Can Zhao, Guohui Li, Ke Xu, Zhongyang Huo

**Affiliations:** Jiangsu Key Laboratory of Crop Genetics and Physiology/Jiangsu Key Laboratory of Crop Cultivation and Physiology/Jiangsu Co-Innovation Center for Modern Production Technology of Grain Crops/Agricultural College, Yangzhou University, No. 88 Daxue South Road, Yangzhou 225009, China; wangwl@yzu.edu.cn (W.W.); lgh@yzu.edu.cn (G.L.); xuke@yzu.edu.cn (K.X.)

**Keywords:** antioxidant capacity, cell wall modification, heat shock protein, dehydrin, defensin

## Abstract

Late spring frost is an important meteorological factor threatening the safe production of winter wheat in China. The young ear is the most vulnerable organ of the wheat plant to spring frost. To gain an insight into the mechanisms underpinning young wheat ears’ tolerance to freezing, we performed a comparative proteome analysis of wheat varieties Xumai33 (XM33, freezing-sensitive) and Jimai22 (JM22, freezing-tolerant) under normal and freezing conditions using label-free quantitative proteomic techniques during the anther connective tissue formation phase (ACFP). Under freezing stress, 392 and 103 differently expressed proteins (DEPs) were identified in the young ears of XM33 and JM22, respectively, and among these, 30 proteins were common in both varieties. A functional characterization analysis revealed that these DEPs were associated with antioxidant capacity, cell wall modification, protein folding, dehydration response, and plant–pathogen interactions. The young ears of JM22 showed significantly higher expression levels of antioxidant enzymes, heat shock proteins, and dehydrin under normal conditions compared to those of XM33, which might help to prepare the young ears of JM22 for freezing stress. Our results lead to new insights into understanding the mechanisms in young wheat ears’ response to freezing stress and provide pivotal potential candidate proteins required for improving young wheat ears’ tolerance to spring frost.

## 1. Introduction

Some transformative changes, such as population growth, urbanization expansion, accelerated land degradation, and climate change, are threatening the future of food security. Wheat is a major staple crop around the world, with global production exceeding 700 million tons per year, feeding more than one-third of the world’s population [1]. Late spring frost (LSF), i.e., subzero temperatures in late spring, is a critical factor threatening the safety of wheat production worldwide, specifically in countries such as the United States [2], Europe [3], Australia [4], and China [5]. LSF may lead to decreased root activity and leaf photosynthetic rate and young ear death and stem damage, resulting in a huge loss of wheat yield [5,6,7]. In Australia, even under optimized management practices, it was estimated that LSFs account for over 10% of the decline in the long-term average wheat production [8]. The frequent occurrence of LSF in the Huanghuai region and the middle and lower reaches of the Yangtze River wheat-growing areas of China have led to a substantial decrease in the final grain yield of 30~50% in severe cases [9]. In the context of global warming, insufficient cold acclimation, an accelerated growth cycle, and increased temperature fluctuations may aggravate the risk and severity of LSF damage in winter wheat [3,10,11].

In China, the harmful effects of LSF on wheat mainly occur in March each year, when plants grow into the jointing stage [12]. This stage is a key period for the rapid growth and differentiation of young wheat ears, which are highly sensitive to freezing temperatures [9]. The death or poor development of young ears is the main reason for wheat yield loss caused by LSF [5,13]. The frost tolerance of young wheat ears declines as development advances through the jointing stage, then drops off particularly suddenly at the point when the anther connective tissue formation phase (ACFP) starts [8]. Therefore, the ACFP is an important stage to evaluate the freezing tolerance of wheat varieties and explore the mechanisms of the young ears’ response to freezing stress.

The mechanisms of young ears responding to low-temperature stress at the meiosis stage have been extensively studied through transcriptomics, metabolomics, lipidomics, and physiological and biochemical analysis [4,13,14]. However, the research on the response mechanisms of young ears to freezing stress at the ACFP is deficient. Recently, Jiang et al. [9] performed a comparative transcriptome analysis of two wheat varieties with different freezing tolerances at the ACFP using RNA-seq data and revealed that the pathways involved in hormone signal transduction, starch, and sucrose metabolism, and circadian rhythm play important roles in young wheat ears’ response to freezing stress. However, transcriptome analysis has limitations because the gene expression levels do not always correspond directly to protein expression due to the post-transcriptional and post-translational modifications [15].

Protein is the main carrier of life activities, controlling the ultimate biological processes. Proteomics is an important research field of the post-genome era, which is a vital link between transcriptomics and metabolomics [15]. The high-throughput proteomics technique has become a powerful approach that provides direct information about how plants respond to stresses at the protein level. A series of studies exist on the proteomics of the spring freezing stress response in wheat leaves. For example, Han et al. [16] analyzed the proteomic changes of wheat leaves under spring freezing using 2-DE and discovered that the enhanced accumulation of the C2H2 zinc finger protein, LEA-related COR protein, Cu/Zn superoxide dismutase, and ascorbate peroxidase played important roles in the mechanisms of response to spring freezing in wheat leaves. Zhang et al. [17] studied the proteomic response in the leaves of two wheat varieties with contrasting freezing tolerance when subjected to spring freezing and found that the different expression patterns of the quinone oxidoreductase-like protein, cold-shock protein, fructose bisphosphate aldolase, rubisco proteins, and ATPase might cause the different freezing tolerances in the two wheat varieties. These studies provide not only global insights into the mechanisms of spring freezing response but also pivotal candidate functional proteins for the genetic improvement in freezing tolerance in wheat leaves. However, studies on the proteome response to spring freezing in young wheat ears at the ACFP are limited.

Therefore, in the present study, we performed a comparative proteomic analysis of the young ears of two wheat varieties with significant differences in freezing tolerance under normal and freezing temperature conditions at the ACFP. The objectives of this study were (1) to explore the mechanisms of the young wheat ears’ response to freezing stress at the protein level and (2) to identify candidate proteins that might play crucial roles in young wheat ears’ freezing tolerance.

## 2. Results

### 2.1. Phenotypic Differences between JM22 and XM33 under Freezing Stress

**Figure 1:**
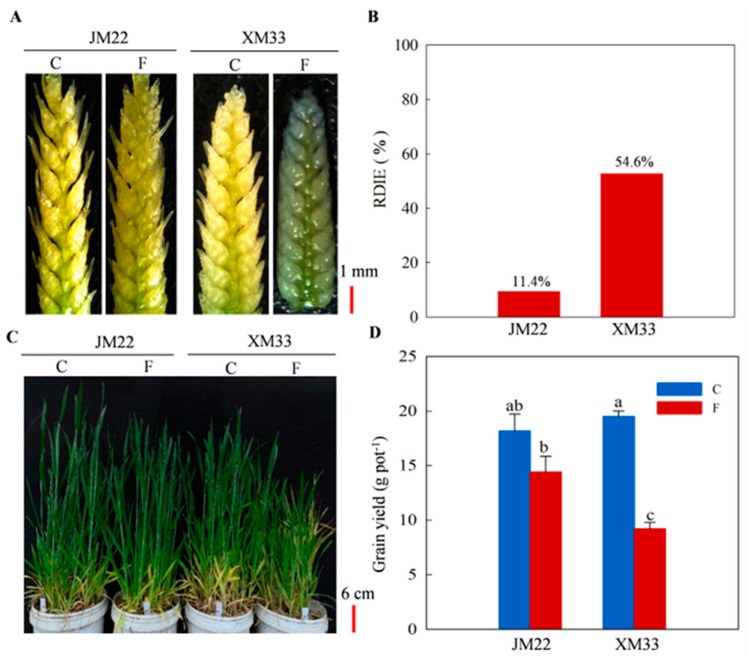
Young ears’ freezing tolerance of JM22 and XM33 varieties at the ACFP. (**A**) Morphological changes in young ears at the end of freezing. (**B**) The ratio of dead/injured ears to the total ears investigated (RDIE). (**C**) Growth performance of wheat plants at the booting stage. (**D**) Grain yield of main stems at maturity. Each value of grain yield is the mean ± SE of three biological replicates, and the different lowercase letters in (**D**) indicate statistically significant differences at the *p* < 0.05 level. C and F indicate control and freezing treatments, respectively.

After 24 h of freezing treatment, severe cell dehydration and shrinkage were visually observed in the young ears of XM33, while the young ears of JM22 did not show obvious injury symptoms (Figure 1A). The ratio of dead/injured ears to the total ears investigated (RDIE) values in XM33 and JM22 caused by freezing treatment were 54.6% and 11.4%, respectively (Figure 1B). As shown in Figure 1C, freezing treatment markedly inhibited the growth of wheat plants, especially of XM33. The grain yield of main stems decreased by 62.9% in XM33 under freezing stress, while JM22 showed less yield reduction (20.9%) compared with XM33 (Figure 1D). These results indicate that the young ears of JM22 are more tolerant to freezing stress than XM33 at the ACFP.

### 2.2. Physiological Response of Young Ears to Freezing Stress

**Figure 2:**
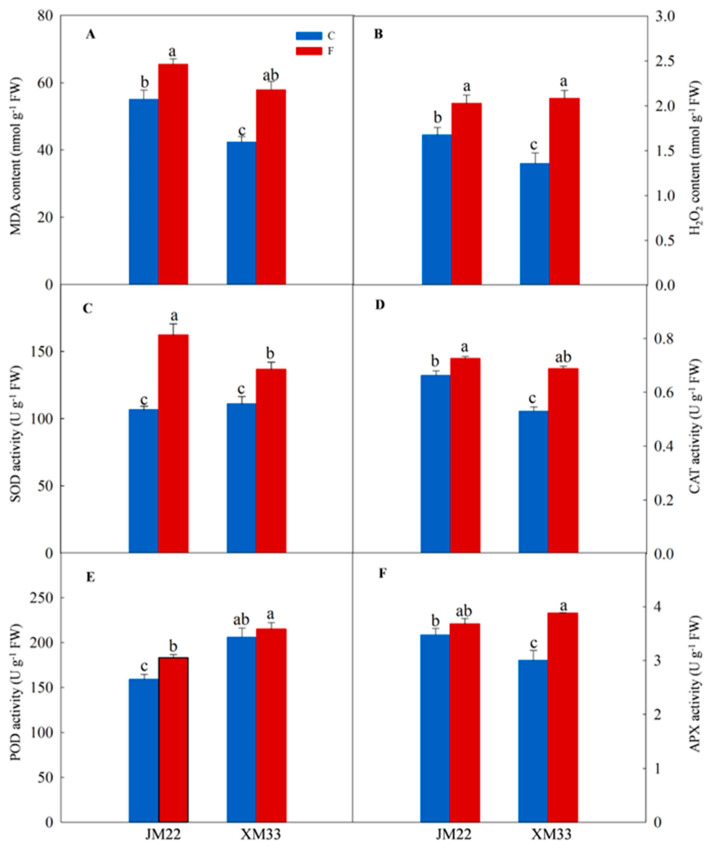
Antioxidant capacity analysis of young wheat ears under normal and freezing conditions. Note: The measurements were taken at the end of the temperature treatment. (**A**,**B**) Contents of malonaldehyde (MDA) and hydrogen peroxide (H_2_O_2_). (**C**–**F**) Activities of superoxide dismutase (SOD), catalase (CAT), peroxidase (POD), and ascorbate peroxidase (APX). C and F indicate control and freezing treatments, respectively. JM22 and XM33 represent freezing−tolerant and freezing−sensitive winter wheat varieties Jimai22 and Xumai 33, respectively. Each value is the mean ± SE of three biological replicates. The different lowercase letters indicate statistically significant differences at the *p* < 0.05 level.

As shown in Figure 2A,B, the accumulation levels of malonaldehyde (MDA) and hydrogen peroxide (H_2_O_2_) were significantly enhanced in the young ears of both varieties, with increases of 18.9% and 21.0% in JM22 and 36.7% and 53.7% in XM33, respectively. Freezing stress significantly increased the activities of superoxide dismutase (SOD) and catalase (CAT) in the young ears of both varieties, with increases of 52.2% and 9.58% in JM22 and 23.32% and 29.9% in XM33, respectively (Figure 2C,D). As compared with normal temperature control, the activity of peroxidase (POD) was enhanced in the young ears of both varieties but only significantly in JM22 under freezing, and the activity of ascorbate peroxidase (APX) was also increased in the young ears of both varieties but only significantly in XM33 under freezing (Figure 2E,F). The young ears of JM22 showed significantly higher activities of CAT and APX than those for XM33 under normal temperature (Figure 2D,F). However, the activity of POD was obviously lower in the young ears of JM22 than that of XM33 under normal temperature (Figure 2E). There was no significant difference in the activity of SOD between the young ears of JM22 and XM33 under normal temperature (Figure 2C).

### 2.3. Protein Identification and Quantification

**Figure 3:**
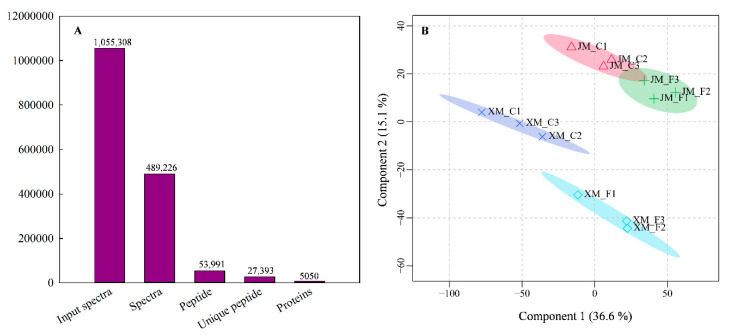
Basic information statistics and partial least squares analysis for label−free quantitative proteomics in young wheat ears. Note: The measurements were taken at the end of the temperature treatment. (**A**) Numbers of spectra, peptides, and proteins identified in young ears. (**B**) Partial least squares (PLS) analysis of the proteome in three biological replicates from freezing−stressed and non−stressed young wheat ears. C and F indicate control and freezing treatments, respectively. JM22 and XM33 represent freezing−tolerant and freezing−sensitive wheat varieties Jimai22 and Xumai 33, respectively.

In this study, a total of 1,055,308 spectra were generated, and 489,226 were matched to known spectra. These detected spectra were assigned to 53,991 peptides, with 27,393 unique peptides (Figure 3A). Finally, 5050 proteins were identified in the young ears of the two varieties at the ACFP (Figure 3A and Table S1). The identified protein molecular weight was mainly distributed between 10 and 60 kDa (Figure S1). The results of the least squares (PLS) analysis showed that three biological replicates of each treatment had good repeatability (Figure 3B). There was an apparent separation in the protein expression profiles of young ears under normal and freezing conditions in both varieties (Figure 3B), indicating that freezing stress caused significant changes in the protein expression profiles of young wheat ears at the ACFP. Additionally, there were striking differences in the protein expression profiles of JM22 and XM33 under both normal and freezing conditions (Figure 3B).

### 2.4. Identification and Analysis of Differently Expressed Proteins

**Figure 4:**
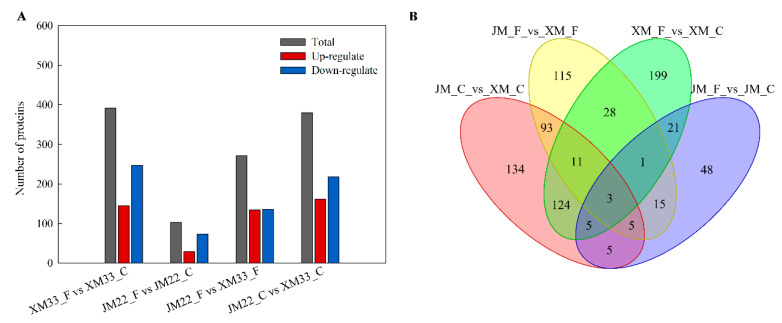
Quantitative and Venn analyses of differently expressed proteins among the different comparative groups. (**A**) Number of differently expressed proteins (DEPs). (**B**) Venn diagrams. C and F indicate control and freezing treatment, respectively. JM22 and XM33 represent freezing−tolerant and freezing−sensitive wheat varieties Jimai22 and Xumai 33, respectively.

We obtained the differentially expressed proteins (DEPs) of the single cultivar under different temperature treatments and the DEPs of two varieties under the same temperature treatments through comparative analysis. As shown in Figure 4A, a total of 807 DEPs were identified in the all−comparative groups, including XM33_F vs. XM33_C, JM22_F vs. JM22_C, JM22_F vs. XM33_F, and JM22_C vs. XM33_C. There were 392 DEPs (145 upregulated and 247 downregulated) in the XM33_F vs. XM33_C group, 103 DEPs (29 upregulated and 74 downregulated) in the JM22_F vs. JM22_C group, 271 DEPs (135 upregulated and 136 downregulated) in the JM22_F vs. XM33_F group and 380 DEPs (162 upregulated, 218 downregulated) in the JM22_C vs. XM33_C group, respectively. The number of DEPs in the XM33_F vs. XM33_C group was significantly higher than that of the JM22_F vs. JM22_C group, further demonstrating that the young ears of XM33 were more sensitive to freezing compared to those of JM22. The downregulated DEPs accounted for 63.0% and 71.8% of the total DEPs in the XM33_F vs. XM33_C and the JM22_F vs. JM22_C groups, respectively. A large number of DEPs existed in the JM22_C vs. XM33_C group, suggesting that the protein expression profiles of XM33 and JM22 young eras were obviously different under normal conditions at the ACFP. The number of DEPs in the young ears of two varieties was decreased by 28.7% under freezing treatment.

There were 30 common DEPs in the XM33_F vs. XM33_C and the JM22_F vs. JM22_C groups, including calcium−dependent protein kinase, heavy metal−associated isoprenylated plant protein, peroxidase, defensin, and so on (Figure 4B and Table 1). Among these proteins, 8 and 19 proteins were upregulated and downregulated in both varieties, respectively. One protein was upregulated in XM33, while it was downregulated in JM22, and two proteins were upregulated in JM22 but downregulated in XM33 (Table 1). There were four common DEPs (A0A3B6IPS7, A0A3B6C0Y8, A0A3B6R8W9, and A0A3B6TGN2) in the XM33_F vs. XM33_C, JM22_F vs. JM22_C, and JM22_F vs. XM33_F groups, and three common DEPs (A0A3B6C0Y8, A0A3B6R8W9, and A0A3B6TGN2) in the all−comparative groups (Figure 4B). These common DEPs may play important roles in young wheat ears’ response to freezing stress.

We randomly chose four common DEPs for expression validation at the transcription level using qRT−PCR. Among them, three genes generally displayed consistent expression patterns with their corresponding proteins; one gene exhibited an opposite trend to its corresponding protein (Figure S2). These results further demonstrate that the changes in mRNA expression do not always accurately reflect corresponding protein levels.

### 2.5. Functional Analysis of the DEPs in Different Comparison Groups

**Figure 5:**
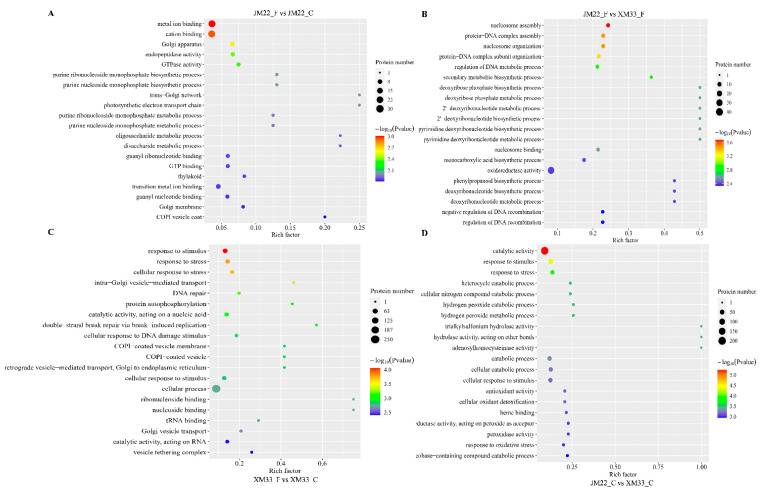
Gene ontology (GO) enrichment analysis of differently expressed proteins in the different comparative groups. (**A**) Top 20 GO terms in the JM22_F vs. JM22_C comparative group. (**B**) Top 20 GO terms in the JM22_F vs. XM33_F comparative group. (**C**) Top 20 GO terms in the XM33_F vs. XM33_C comparative group. (**D**) Top 20 GO terms in the JM22_C vs. XM33_C comparative group. C and F indicate control and freezing treatments, respectively. JM22 and XM33 represent freezing-tolerant and freezing-sensitive wheat varieties Jimai22 and Xumai 33, respectively.

Gene ontology (GO) enrichment analysis was used to functionally characterize the DEPs in different comparative groups. In the JM22_F vs. JM22_C group, the DEPs were significantly (*p* < 0.05) enriched in 54 GO terms, including “metal ion binding”, “cation binding”, “Golgi apparatus”, “endopeptidase activity”, and so on (Figure 5A). The DEPs in the XM33_F vs. XM33_C group significantly (*p* < 0.05) matched 222 GO terms. The top 20 enriched terms are exhibited in Figure 5C, including “response to stimulus”, “response to stress”, “cellular response to chemical stimulus”, “intra−Golgi vesicle−mediated transport”, and so on (Figure 5C). The obvious difference in the enriched GO terms between the XM33_F vs. XM33_C and the JM22_F vs. JM22_C groups suggests that there are significant differences in the freezing response mechanisms of the two varieties. In the JM22_F vs. XM33_F group, the top 20 significantly (*p* < 0.05) enriched GO terms for the DEPs were “nucleosome assembly”, “nucleosome organization”, “protein−DNA complex assembly”, “protein−DNA complex subunit organization”, and so on (Figure 5B). The top 20 enriched terms for the DEPs in the JM22_C vs. XM33_C group were “catalytic activity”, “response to stimulus”, “response to stress”, “cellular nitrogen compound catabolic process”, and so on (Figure 5D), suggesting that the young ears of JM22 at the ACFP may cope better with potential environmental stresses than those of XM33.

### 2.6. Key Proteins Associated with Freezing Stress Response in the Young Ears

In order to deeply explore the mechanisms of young wheat ears responding to freezing stress, we further analyzed the functions of the DEPs in each comparative group based on the results of GO enrichment analysis.

#### 2.6.1. Antioxidant-Related Proteins

In the XM33_F vs. XM33_C group, there were 12 DEPs (10 upregulated and 2 downregulated) related to antioxidant activity, includingPOD, dehydroascorbate reductase (DHAR), glutathione transferase (GST), and so on (Table 2). However, only four DEPs were associated with antioxidant activity in the JM22_F vs. JM22_C group, and their expression levels were all downregulated. In the JM22_F vs. XM33_F group, a total of nine DEPs belong to antioxidant enzymes, of which three were upregulated, and six were downregulated. However, there were 14 DEPs related to antioxidants (10 upregulated and 4 downregulated) in the JM22_C vs. XM33_C group, including APX, POD, DHAR, and so on (Table 2).

#### 2.6.2. Heat Shock Proteins

As shown in Table 3, eight DEPs were annotated as heat shock proteins (HSPs) in the XM33_F vs. XM33_C group, of which seven were upregulated and one was downregulated. However, there were no DEPs belonging to HSPs in the JM22_F vs. JM22_C group. There were five HSPs (three upregulated and two downregulated) and five HSPs (four upregulated and one downregulated) in the JM22_F vs. XM33_F group and the JM22_C vs. XM33_C group, respectively (Table 3).

#### 2.6.3. Cell Wall−Modifying Related Proteins

As shown in Table 4, the expression level of polygalacturonase inhibitor−like protein (A0A3B6TGN2) was significantly upregulated in the JM22_F vs. JM22_C and the XM33_F vs. XM33_C groups. The expression levels of pectinesterase inhibitor 7−like protein (A0A1D5V3A3) and xyloglucan endotransglucosylase/hydrolase protein (A0A3B6TWG3) were significantly upregulated, while the expression level of alpha−1,3−arabinosyltransferase protein (A0A3B6TI25) was downregulated in the XM33_F vs. XM33_C group. One cinnamyl−alcohol dehydrogenase protein (A0A3B6BYY2) was downregulated in the JM22_F vs. JM22_C group and upregulated, together with one cinnamyl−alcohol dehydrogenase protein (A0A3B6CBN0) in the XM33_F vs. XM33_C group. In addition, one cinnamoyl-CoA reductase protein (A0A3B6RK39) was downregulated in the JM22_F vs. JM22_C group but not in the XM33_F vs. XM33_C group. Moreover, one germin−like protein (A0A3B6H1P4) was obviously upregulated in the XM33_F vs. XM33_C group.

One polygalacturonase inhibitor−like protein (A0A3B6TGN2) and one expansin−B4−like protein (A0A3B6KU54) were upregulated in the JM22_F vs. XM33_F group. In addition, two endoglucanase proteins (A0A3B6IKN1 and A0A3B5Y507), two xyloglucan endotransglucosylase/hydrolase proteins (Q56TP1 and A0A3B6TWG3), one probable polygalacturonase isoform X1 protein (A0A3B6TGM9), and one cinnamyl alcohol dehydrogenase protein (A0A3B6CBN0) were downregulated in the JM22_F vs. XM33_F group. In the JM22_C vs. XM33_C group, one endoglucanase (A0A3B5Y507) and one UDP−arabinopyranose mutase (A0A3B6B5P1) were significantly upregulated, and one xyloglucan endotransglucosylase/hydrolase (A0A3B6DED8), one alpha−1,3−arabinosyltransferase (A0A3B6TI25), and one beta−D−xylosidase 4-like protein (A0A3B6DMR3) were obviously downregulated (Table 4).

## 3. Discussion

### 3.1. Enhancing Antioxidant Capacity Was Beneficial for Young Wheat Ears’ Freezing Tolerance

Reactive oxygen species (ROS) are byproducts of aerobic metabolic processes (such as electron transport and photorespiration). Due to ROS being highly reactive and toxic, their over−accumulation can cause oxidative damage to biological macromolecules (lipids, proteins, nucleic acids, etc.) and eventually lead to cell death [18]. It has been documented that freezing stress may induce the over-accumulation of ROS and cause oxidative destruction of cells in many plant species, including wheat [6]. In the present study, freezing stress significantly increased the accumulation level of H_2_O_2_ and MDA in young wheat ears (Figure 2A,B), indicating that freezing−induced oxidative damage to young ears of wheat. These results are well consistent with those of Jiang et al. [9], who found that low−temperature stress (chilling or freezing) obviously enhanced the level of MDA in young wheat ears. Antioxidant enzymes play a crucial role in scavenging ROS and maintaining redox balance in wheat leaves under freezing stress [6,19]. In the present study, the freezing−induced upregulated DEPs in the young ears of XM33, which were significantly enriched in the GO terms of “cell redox homeostasis”, “ascorbate−glutathione cycle”, and “hydrogen peroxide metabolic process” (Figure S3). The expression levels of antioxidant enzymes, including POD, DHAR, and GST, were significantly upregulated in the young ears of XM33 under freezing (Table 2). Meanwhile, the activities of SOD, CAT, and APX were obviously increased in the young ears of XM33 under freezing (Figure 2). These results suggest that improving the antioxidant capacity is an important physiological mechanism for young wheat ears responding to freezing stress. However, the freezing−induced upregulated DEPs in the young ears of JM22 did not become enriched in antioxidant−related terms in this study (Figure S4). However, it was worth noting that the upregulated DEPs in the young ears of JM22 were enriched in the GO terms of “antioxidant activity”, “antioxidant activity”, “hydrogen peroxide catabolic process”, and “peroxidase activity” compared with those of XM33 under normal conditions (Figure S5). The expression levels of many antioxidant enzymes, including POD, DHAR, and APX, were strikingly upregulated in the young ears of JM22 compared with those of XM33 under normal conditions (Table 2). In addition, the activities of CAT and APX in the young ears of JM22 were significantly higher than those in XM33 under normal conditions (Figure 2D,F). These results indicate that the young ears of JM22 had a stronger antioxidant capacity than those of XM33 under normal conditions. It was speculated that the young ears of JM22 may clear ROS more quickly than those of XM33 when subjected to freezing stress. The lower increments of H_2_O_2_ and MDA content in the young ears of JM22 under freezing may provide evidence for this speculation (Figure 2A,B). Therefore, the difference in antioxidant capacity may be one of the reasons why the young ears of JM22 were more tolerant to freezing stress than those of XM33.

### 3.2. Heat Shock Proteins Were Involved in Young Wheat Ears Coping with Freezing Stress

Heat shock proteins (HSPs) are not only highly expressed in plants under heat stress but also are produced in response to various biotic and abiotic stresses [20]. It was established that HSPs could serve as molecular chaperones in the processes of protein transport, folding, and assembly/disassembly, which may go wrong and thereby lose their potential functions during stress conditions [21]. In this study, the freezing−induced DEPs in the young ears of XM33 were enriched in the GO term of “misfolded protein binding” (Figure S3), indicating that freezing stress caused severe protein misfolding in the young ears of XM33. Meanwhile, the expression levels of seven HSPs were significantly upregulated in the young ears of XM33 under freezing stress compared to its normal temperature control (Table 3), which may be beneficial for alleviating protein misfolding caused by freezing. In addition, the young ears of JM22 exhibited higher expression levels of HSPs compared to the young ears of XM33 under both normal and freezing conditions (Table 3). These results indicate that HSPs may play important roles in the young wheat ears responding to freezing stress. Our previous study found that freezing stress significantly upregulated the expression levels of *HSP70* and its upstream transcription factor encoding gene *HSF3* in wheat leaves, and enhancing their expression levels effectively improved the tolerance of wheat leaves to freezing [6]. In this present study, the expression levels of two HSP70 proteins (A0A3B6JIR3 and A0A3B6KKG9) in the young ears of XM33 were significantly upregulated under freezing stress (Table 3). Thus, it appears that HSP70 could be used as a candidate protein for improving later spring frost tolerance of wheat plants.

### 3.3. Changes in Cell Wall Traits Participated in the Response of Young Wheat Ears to Freezing Stress

Ice crystallization takes place in the apoplast spaces in plants under natural freezing stress [22]. Cell walls play a crucial role in resisting mechanical damage caused by ice crystals and preventing cell deformation and collapse induced by dehydration under freezing [23]. Previous studies found that cell wall remodeling was involved in cold acclimation-induced freezing tolerance in many species [24,25]. However, only limited information is available on how changes in cell wall composition and structure affect plant freezing tolerance, especially in wheat. Polygalacturonase and pectinesterase are important in catalyzing the degradation of pectin [23,26]. In this study, the expression level of polygalacturonase inhibitor−like protein (A0A3B6TGN2) was significantly upregulated in the young ears of both varieties under freezing stress. In addition, the expression level of pectinesterase inhibitor 7−like protein (A0A1D5V3A3) was significantly upregulated in the young ears of XM33 under freezing stress (Table 4). These results indicate that the synthesis of pectin was promoted, while its degradation was inhibited in young wheat ears under freezing stress. Baldwin et al. [24] found that freezing tolerance in peas was related to the increase in pectin content and the degree of methyl esterification of pectins. Pectin may contribute to the disruption of ice formation, the maintenance of hydration status, and cell wall flexibility and rigidity under freezing stress [24,27]. Moreover, the young ears of JM22 showed a higher expression level of polygalacturonase inhibitor−like protein (A0A3B6TGN2) and a lower expression level of polygalacturonase (A0A3B6TGM9) than the young ears of XM33 under freezing stress (Table 4). Collectively, increasing cell wall pectin content may be an important mechanism for withstanding freezing stress in young wheat ears.

Cinnamoyl−CoA reductase (CCR) and cinnamyl−alcohol dehydrogenase (CAD) are key enzymes in the lignin biosynthesis pathway, which catalyzes the first step committed to the lignin metabolism branch pathway and the last step in the synthesis of lignin monomer, respectively [28,29]. In this present study, the expression level of CAD (A0A3B6CBN0) was significantly upregulated in the young ears of XM33, whereas the expression levels of CAD (A0A3B6BYY2) and CCR (A0A3B6RK39) were strikingly downregulated in the young ears of JM22 (Table 4). These results suggest that the synthesis of lignin in young ears was promoted in the freezing−sensitive variety but was inhibited in the freezing−tolerant variety. In addition, the young ears of JM22 showed a lower expression level of CAD (A0A3B6CBN0) compared with the young ears of XM33 under freezing stress (Table 4). Lignin is a major component of plant cell walls, which plays an important role in reducing water permeability and increasing cell wall stiffness [30]. It has been recorded that the lignin content was negatively associated with plant freezing tolerance [25]. A low lignin content may be beneficial for maintaining the elasticity of cell walls, which should help alleviate mechanical damage caused by extracellular ice crystals and cell dehydration under freezing conditions [31]. Therefore, the differential response in lignin metabolism may be one of the reasons for the difference in freezing tolerance between the young ears of JM22 and XM33.

Xyloglucan, xylan, and arabinoxylan are the major hemicelluloses present in plant cell walls. Xyloglucan endotransglucosylase/hydrolase (XTH) has a dual role in plant cell wall modification by either hydrolyzing or remodeling xyloglucans [32]. Takahashi et al. [33] found that the *Arabidopsis XTH19* mutant showed obviously reduced freezing tolerance compared to the wild type. In this study, freezing stress significantly increased the expression level of XTH (A0A3B6TWG3) in the young ears of XM33 (Table 4), suggesting that the XTH activity was also associated with the freezing tolerance of young wheat ears. However, the young ears of JM22 showed lower expression levels of XTH (Q56TP1 and A0A3B6TWG3) compared with those of XM33 under freezing stress. Thus, the role of XTH in young wheat ears’ response to freezing stress needs further investigation. Xylan arabinosyl−transferase (XAT) is a key enzyme that catalyzes the glycosylation of xylan to synthesize arabinoxylan [34]. In this present study, the expression level of XAT (A0A3B6TI25) was strikingly decreased in the young ears of XM33 (Table 4). Chen et al. [34] reported that a rice *XAT* knockout mutant exhibited decreased arabinose content. Arabinose is a crucial component that mediates the linkage between xylan and lignin. It was speculated that the reduction in XAT activity may help the cell walls maintain their flexibility to alleviate freezing stress−induced mechanical injury.

### 3.4. Other Proteins May Contribute to Enhance Freezing Tolerance in Young Wheat Ears

Plant defensins are known as a class of pathogenesis−related (PR) proteins, which usually have both antifungal and anti−freezing activities in plants [35]. Isobe et al. [36] reported that TAD1 (*Triticum aestivum* defensin 1), a plant defensin, was specifically induced by cold acclimation treatment in winter wheat leaves. There was no significant change in the expression level of TAD1 (Q8L698) in the young ears of both varieties under freezing stress compared with their normal temperature control in this study (Tables S2 and S3). However, the expression level of TAD1 in the young ears of JM22 was significantly higher (2.05-fold) than that in the young ears of XM33 under freezing stress (Table S4). In addition, the expression level of another defensin (W5AMD3, named PDF10) was significantly upregulated (37.1-fold) in the young ears of JM22 under freezing stress, while the expression of PDF10 was obviously inhibited (0.606-fold) in the young ears of XM33 under freezing stress (Tables S2 and S3). It is worth noting that the expression level of PDF10 in the young ears of JM22 was strikingly lower (0.0048-fold) than that in the young ears of XM33 under normal temperature conditions (Table S5). Hiilovaara-Teijo et al. [37] found that only the PR proteins induced by cold temperature exhibited antifreeze activity. Thus, the PDF10 expressed in the young ears of XM33 under normal temperatures may not have antifreeze activity. It was revealed that the expression pattern of defensins (especially PDF10) under freezing was closely related to the freezing tolerance of young wheat ears.

Heavy metal−associated isoprenylated plant proteins (HIPPs) are characterized by the presence of one or more heavy metal−associated (HMA) domains and C−terminal prenylation/farnesylation sites [38]. Previous studies have reported that HIPPs are involved in heavy metal homeostasis and adaptation to biotic and abiotic stresses in plants [39,40]. In the present study, the expression levels of two HIPP proteins (A0A3B6HTB1 and W5B397) were commonly upregulated (1.51-fold and 1.78-fold in XM33, 1.52-fold and 1.76-fold in JM22, respectively) in the young ears of both varieties under freezing stress compared to their normal temperature controls (Table 1, Tables S2 and S3), indicating that HIPPs may play an important role in young wheat ears’ response to freezing. The upregulation of HIPP expression induced by low temperature was reported by several studies [39,40]. However, their biological functions are still largely unknown [38]. Barth et al. [41] found that the HMA domain of HIPPs could interact with the zinc finger homeodomain of stress−related transcription factors, suggesting that HIPPs may play a role in transcriptional regulation.

It has been well documented that the accumulation of dehydrins is associated with the development of freezing tolerance [42,43]. Dehydrins have the functions of being a protein cryoprotectant, antifreeze, and ROS scavenger in plants under freezing stress [22]. In the present study, the expression level of one dehydrin (T1VYS7) was significantly upregulated (1.71-fold) in the young ears of XM33 under freezing stress (Table S3). In addition, the young ears of JM22 showed a significantly higher expression level of T1VYS7 (1.69-fold) than the young ears of XM33 under normal temperature conditions (Table S5). Thus, T1VYS7 may play a positive role in the response of young wheat ears to freezing stress.

### 3.5. Maintaining Transcriptional Activity Was a Crucial Biological Basis for Freezing Tolerance in Young Wheat Ears

Environmental stresses can cause profound changes in the plant transcriptome. For example, cold−regulated genes have been estimated to constitute approximately 4% to 20% of the genome in *Arabidopsis* [44]. In this study, freezing stress−induced significant changes in the expression levels of 807 proteins, accounting for 16.0% of the total proteins identified in young ears (Figure 4A). The number of downregulated proteins was obviously higher than that of upregulated proteins under freezing stress in both varieties, indicating that freezing stress generally inhibited protein expression in young wheat ears. The freezing−induced downregulated DEPs in the young ears of XM33 were significantly enriched in the GO terms of “catalytic activity, acting on a nucleic acid”, “nucleic acid metabolic process”, “DNA−templated DNA replication”, “double−strand break repair via break−induced replication”, “Golgi vesicle transport”, and so on (Figure S6), suggesting that the decreased expression levels of proteins involved in nucleic acid metabolism and protein synthesis under freezing resulted in the sensitivity to freezing of the young ears of XM33. Interestingly, the freezing−induced upregulated DEPs in the young ears of JM22 were significantly enriched in the GO terms of “transcription regulator complex”, “regulation of transcription by RNA polymerase II”, “ncRNA processing”, and so on (Figure S4). Moreover, the upregulated DEPs in the young ears of JM22 were significantly enriched in the GO terms of “nucleosome assembly”, “nucleosome organization”, and “chromatin remodeling” compared to the young ears of XM33 under freezing stress (Figure S7). These results revealed that the genome in the young ears of JM22 had stronger plasticity than that of XM33 and thus could maintain higher transcriptional activity under freezing. This may be an important biological basis for the tolerance of young ears of JM22 to freezing stress.

## 4. Materials and Methods

### 4.1. Plant Materials, Growing Conditions, and Temperature Treatments

This experiment was conducted at the experimental station of Yangzhou University, Yangzhou, Jiangsu Province, China, during the wheat growing season of 2021–2022. Two winter wheat (*Triticum aestivum* L.) varieties, Jimai22 (JM22, freezing−tolerant) and Xumai33 (XM33, freezing−sensitive), were selected as experimental materials. The seeds were planted in plastic pots (25 cm in diameter × 22 cm in height) filled with 9.5 kg of clay soil. The planting density was 10 plants per pot. Before the pots were filled, the soil was homogenously mixed with 1.0 g urea, with 1.5 g of KH_2_PO_4_ per pot. At the regreening stage and the booting stage, 0.5 g of urea per pot was applied with irrigation water, respectively. The plants were grown outdoors until they reached the ACFP. The local daily mean temperature and precipitation during the study period are shown in Figure S8 (xihe-energy.com). Wheat plants planted in pots can grow normally outdoors under local temperature conditions, and six holes were drilled into the bottom of each pot to prevent waterlogging. Artificial water replenishment was performed whenever the pot soil was becoming dry, and the amount of irrigation in each pot was consistent.

The morphologies of the ears of the main stems in two varieties were dynamically monitored, using a microscope to judge their development stage. Plants of each variety were randomly divided into two groups when the ears of their main stems developed to the ACFP and were transferred into an artificial climate chamber (Eshengtaihe Co., Ltd., Beijing, China; temperature control range: −10~45 °C, temperature constancy: ± 0.5–1.0 °C, temperature uniformity: ±1 °C; humidity control range: 50–95% RH; illumination intensity: 150–500 μmol m^−2^ s^−1^; L × W × H: 5 m × 2 m × 3 m) for temperature treatment. One group was transferred into a chamber with a temperature of 15 °C/5 °C (day/night, set according to the ambient temperature) as the control treatment (C); another group was transferred into a chamber for the freezing treatment (F). The freezing treatment procedure involved cooling the chamber temperature at a rate of 2 °C·h^−1^, and then holding it at −4 °C for 24 h. The young ears of the main stem were sampled at the end of the freezing treatment for further physiological and proteomic analyses. The samples were named JM22_C (young ears of JM22 at normal temperature), JM22_F (young ears of JM22 treated at freezing temperature), XM33_C (young ears of Xumai33 at normal temperature), and XM33_F (young ears of Xumai33 at freezing temperature). After freezing treatment, the chamber temperature was increased to 4 °C for 12 h, and then the remaining plants were moved out and grown under natural conditions until harvest. During the temperature treatment, the photosynthetically active radiation (PAR) of the chambers was set at 300 μmol m^−2^ s^−1^ with a photoperiod of 10 h, and the relative air humidity was kept at approximately 70%.

### 4.2. Young Ears’ Freezing Tolerance Determination

Seven days after the freezing treatment, the degree of freezing damage to the ears was investigated with light microscopy (Leica S8APO, Leica Microsystems, Wetzlar, Germany). Any wilted and whitish parts in the ears were judged to have been injuries from freezing. At least 30 main stem ears of each treatment were investigated. The ratio of dead/injured ears to the total ears investigated (RDIE) was used as a direct index to evaluate young ears’ freezing tolerance [8]. For grain yield determination, three pots of each treatment were randomly selected, and the main stems of plants in each pot were marked before the temperature treatments. Spikes of the main stems from each pot were separately harvested at maturity, followed by manual threshing to obtain the grain. The loss of grain yield was used to reflect the freezing tolerance of young ears at the ACFP.

### 4.3. Physiological Parameters Measurement

The contents of malonaldehyde (MDA), hydrogen peroxide (H_2_O_2_) and the activities of superoxide dismutase (SOD; EC 1.15.1.1), catalase (CAT; EC 1.11.1.6), peroxidase (POD; EC 1.11.1.7), and ascorbate peroxidase (APX; EC1.11.1.11) were determined at the end of the temperature treatments. The measurement of MDA content and antioxidant enzyme activities was performed as described by our previous study [6]. The H_2_O_2_ content was measured using assay kits from Jiancheng Bioengineering Institute, China.

### 4.4. Gene Expression Analysis

The total RNA extraction, cDNA synthesis, and qRT−PCR were carried out according to the method described by Wang et al. [45]. The specific primers of the corresponding protein genes are listed in Table S6. The relative expression levels of genes were calculated according to the 2^−ΔΔCt^ method, using the *Actin* gene as the reference gene. Three biological replicates and three technical replicates were performed for each gene.

### 4.5. Total Protein Extraction and Digestion

The total protein was extracted using the plant total protein extraction kit (Bangfei Bioscience Co., Ltd., Beijing, China) according to the manufacturer’s instructions. Briefly, 0.2 g (fresh weight) of young ears were ground into powder in liquid nitrogen using a mortar and pestle. The powder was added to a solution of PE solution A, and the resulting suspension was mixed thoroughly with vortexing. To the mixture was added an equal volume of PE solution B followed by incubation on ice for 1 h. After centrifugation at 15,000× *g* for 20 min at 4 °C, the supernatant was collected, and then PE solution C was added. For this procedure, the collections were restored at −20 °C overnight and centrifugated at 15,000× *g* for 15 min at 4 °C. The remaining pellet was washed twice with precooled acetone and resuspended in lysis buffer PE solution D. The protein concentrations were determined using the protein quantification kit (Dingguo Changsheng, Beijing, China) with bovine serum albumin as the standard, and the quality of the protein preparation was further evaluated via SDS−PAGE with Coomassie Brilliant Blue R−250 staining. The protein digestion was performed primarily following the filter−aided sample preparation (FASP) protocol as described by Wisniewski et al. [46]. For all samples, three biological replicates were performed.

### 4.6. LC–MS/MS Analysis

LC–MS/MS analysis was performed on a Q Exactive HF−X mass spectrometer (Thermo Fisher Scientific, Waltham, MA, USA) that was coupled to HPLC (Easy−nLC/Ultimate 3000, Thermo Fisher Scientific, Waltham, MA, USA). The digested peptide mixtures were loaded onto a C18−reversed phase analytical column (Thermo Fisher Scientific, Waltham, MA, USA) in buffer A (0.1% formic acid) and separated with a linear gradient of buffer B (80% acetonitrile and 0.08% formic acid) at a flow rate of 600 nl/min. The linear gradient is shown in Table S7. MS data were dynamically chosen from among the most abundant precursor ions from the survey scan (350–1550 *m*/*z*) for HCD fragmentation. The MS1 scans were acquired at a resolution of 120,000 with an AGC target of 3e6 and a maxIT of 20 ms. The MS2 scans were acquired at a resolution of 15,000 at *m*/*z*110 with a maxIT of 30 ms, and the isolation width was 1.6 *m*/*z*. The normalized collision energy was 27 eV.

### 4.7. Sequence Database Search

The resulting MS/MS data were searched using the MaxQuant engine (v.2.1.0.0) against UniProt *Triticum aestivum* L. (proteome ID: UP000019116; containing 130,673 sequences). Trypsin/P was specified as the cleavage enzyme, allowing up to 2 missing cleavages. The search included a fixed modification of carbamidomethyl cysteine as well as variable modifications of methionine oxidation and *N*−terminal acetylation. The peptide mass tolerance and fragment mass tolerance were set to ±4.5 ppm and 20 ppm, respectively. The cutoff of the global false discovery rate (FDR) for peptide and protein identification was set to 0.01.

### 4.8. Data Analysis

In the quantitative analysis, each confident protein identification involved at least two unique peptides, and the proteins containing at least two or more quantitative values in one set of samples (three biological repeats) were retained. The protein abundance was calculated on the basis of the normalized spectral protein intensity (LFQ intensity). Missing values imputation of protein intensities were performed from a normal distribution using Perseus software (v.2.0.5.0). The ratio of the mean LFQ intensity between the two samples indicates the protein fold−change (FC) value. The available quantitative proteins were regarded as differentially expressed proteins (DEPs) with at least a 1.5−FC and a *p*-value less than 0.05 (up, FC ≥ 1.5 and *p* < 0.05; down, FC ≤ 0.667 and *p* < 0.05). The identified DEPs were categorized using the Gene Ontology (GO) database (http://www.geneontology.org/, URL (accessed on 28 June 2022)), and the hypergeometric test was used to find significant enrichment GO terms by comparing differentially expressed proteins with the overall identified proteins as the background. The visualized results from Venn, partial least squares (PLS), and GO enrichment were obtained using an R script.

The grain yield and physiological assay results were analyzed using SPSS 18.0 software (SPSS Inc., IL, USA), and Duncan’s multiple range test was used to analyze the statistically significant differences at the *p* < 0.05 level.

## 5. Conclusions

**Figure 6:**
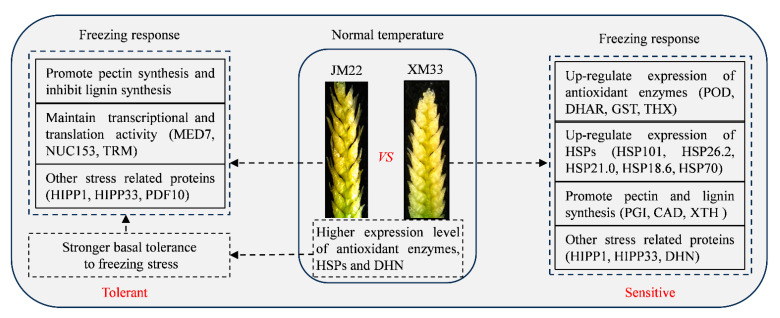
Summary showing the proposed mechanisms of young ears responding to freezing stress in JM22 and XM33. POD, peroxidase; DHAR, dehydroascorbate reductase; GST, glutathione transferase; THX, thioredoxin; HSPs, heat shock proteins; PGI, polygalacturonase inhibitor; CAD, cinnamyl−alcohol dehydrogenase; XTH, xyloglucan endotransglucosylase/hydrolase; HIPP, heavy metal−associated isoprenylated plant protein; DHN, dehydrin; MED; mediator of RNA polymerase II transcription; NUC; NUC153 domain−containing protein; TRM, tRNA (guanine(37)−N1)−methyltransferase; PDF, plant defensin. JM22 and XM33 represent freezing−tolerant and freezing−sensitive wheat varieties Jimai22 and Xumai 33, respectively.

In summary, comparative analyses of proteomics provided comprehensive insights into the overall and variety−specific mechanisms underlying responses to freezing in the young ears of two different wheat varieties (Figure 6). The accumulation of proteins, including antioxidant enzymes, HSPs, and dehydrin, was crucial for the young ears of XM33 in their response to freezing stress. The young ears of JM22 showed higher expression levels of antioxidant enzymes, HSPs, and dehydrin than the young ears of XM33 under normal conditions, indicating that the young ears of JM22 may respond to freezing faster and more strongly than the young ears of XM33. Increasing the pectin content and decreasing the lignin content to improve the flexibility of cell walls may be an important physiological mechanism for young wheat ears responding to freezing stress. The differential expression patterns of defensins and lignin metabolic enzymes in two varieties of young wheat ears under freezing may partly be attributed to their significant differences in freezing tolerance. To the best of our knowledge, this is the first report to study the mechanisms of young wheat ears in response to freezing stress via proteomic means at the ACFP. The differential proteins identified in this study should be used as candidate proteins to improve later spring frost tolerance of young wheat ears in the future. However, further research is required to deeply explore the detailed mechanisms of the responses of young wheat ears to freezing stress.

## Figures and Tables

**Table 1:** Common differentially expressed proteins in the groups of XM33_F vs. XM33_C and JM22_F vs. JM22_C.

Protein ID	Protein Description	XM33_F vs. XM33_C	JM22_F vs. JM22_C
A0A3B6HTB1	Heavy metal−associated isoprenylated plant protein 33−like	up	up
W5B397	Heavy metal−associated isoprenylated plant protein 1	up	up
A0A3B6TCM5	NUC153 domain−containing protein	up	up
A0A3B6KR91	GLTP domain−containing protein	up	up
A0A3B6TGN2	Polygalacturonase inhibitor−like	up	up
W5BGU5	Mediator of RNA polymerase II transcription subunit 7	up	up
A0A3B6C0Y8	DIBOA−glucoside dioxygenase BX6−like	up	up
A0A3B6FJ71	Lactamase_B domain−containing protein	up	up
A0A3B6CBP0	Coatomer subunit epsilon	down	down
A0A3B5YVU9	Methyltransferase	down	down
A0A3B6TGT6	Curvature thylakoid 1A, chloroplastic−like protein	down	down
A0A3B6RGZ0	Serinethreonine−protein phosphatase 4 regulatory subunit 2−A−like	down	down
W4ZSG9	Thioredoxin−like protein YLS8	down	down
A0A1D6RNK4	E3 ubiquitin−protein ligase ARI8	down	down
A0A3B6H1K4	Coatomer subunit zeta	down	down
Q36813	NAD(P)H dehydrogenase subunit H	down	down
Q76ME3	ADP−ribosylation factor	down	down
A0A3B5Y6Q2	SAC domain−containing protein	down	down
A0A3B6TKQ8	COP9 signalosome complex subunit 3−like isoform X2	down	down
Q6KCK6	Putative calcium−dependent protein kinase	down	down
W5EJ68	Protein kinase domain-containing protein	down	down
A0A3B6JPZ5	Uncharacterized protein	down	down
A0A3B6IPS7	Uncharacterized protein	down	down
A0A3B6N1N4	Uncharacterized protein	down	down
A0A3B6KJM2	Uncharacterized protein	down	down
A0A077RWW5	Uncharacterized protein	down	down
A0A3B5XX28	Uncharacterized protein	down	down
A0A3B6LPS5	Peroxidase	up	down
W5AMD3	Defensin PDF10	down	up
A0A3B6R8W9	Uncharacterized protein	down	up

**Table 2:** Differentially expressed antioxidant-related proteins in different comparative groups.

Groups	Protein ID	Protein Description	Fold-Change
XM33_F vs. XM33_C	A0A3B6IUJ0	GST N−terminal domain−containing protein	1.887
	A0A3B6LPS5	Peroxidase	1.853
	A0A3B5XVD9	Probable glutathione S−transferase−cytosolic	1.839
	A0A3B5YRG4	Probable glutathione S−transferase DHAR10, cytosolic	1.754
	A0A3B6JN31	Glutathione transferase	1.713
	A0A3B6B868	Thioredoxin domain−containing protein	1.705
	A0A3B6ED41	2−alkenal reductase (NADP(+) dependent) −like	1.599
	A0A3B6DBD5	Thioredoxin	1.588
	A0A3B6GVH3	Glutaredoxin−dependent peroxiredoxin	1.584
	A0A3B6SJF8	Glutaredoxin−dependent peroxiredoxin	1.547
	W4ZSG9	Thioredoxin−like protein YLS8	0.495
	A0A3B5YYX5	Glutathione transferase	0.342
JM22_F vs. JM22_C	A0A3B6DL50	Thioredoxin domain−containing protein	0.635
	A0A3B6SS74	L−ascorbate oxidase homolog	0.601
	W4ZSG9	Thioredoxin−like protein YLS8	0.593
	A0A3B6LPS5	Peroxidase	0.243
JM22_F vs. XM33_F	A0A3B5YT02	Peroxidase	4.156
	A0A3B6CJF7	Peroxidase	2.589
	A0A3B5ZRA7	Peroxidase	2.275
	A0A3B6LUE2	Glutathione transferase	0.611
	A0A3B6SS74	L−ascorbate oxidase homolog	0.515
	A0A3B6JN31	Glutathione transferase	0.492
	F1DKC1	Catalase	0.445
	A0A3B6TWE0	Peroxidase	0.365
	A0A3B6QC91	Peroxidase	0.250
JM22_C vs. XM33_C	A0A3B5YT02	Peroxidase	4.789
	A0A3B6LPS5	Peroxidase	3.526
	A0A3B5ZRA7	Peroxidase	3.427
	A0A3B6TQJ1	Thioredoxin domain−containing protein	3.021
	A0A3B6CJF7	Peroxidase	2.500
	A0A3B6IRX6	L−ascorbate peroxidase	2.222
	A0A3B5YRG4	Probable glutathione S−transferase DHAR1, cytosolic	1.965
	A0A3B6JL78	L−ascorbate peroxidase	1.701
	A0A3B6AYZ6	L−ascorbate peroxidase	1.536
	A0A1D6D173	Glutaredoxin domain−containing protein	1.522
	A0A3B6DNN1	Peroxidase	0.597
	F1DKC1	Catalase	0.537
	A0A3B6KNG3	Thioredoxin−like fold domain−containing protein	0.290
	A0A3B6QC91	Peroxidase	0.281

**Table 3:** Differentially expressed heat shock proteins in different comparative groups.

Groups	Protein ID	Protein Description	Fold-Change
XM33_F vs. XM33_C	A0A3B6LMB4	DEHY	2.498
	A0A3B6PT83	18.6 kDa class III heat shock protein	2.472
	A0A3B5ZYT4	Heat shock protein 101	2.133
	A0A3B6RDZ6	26.2 kDa heat shock protein−mitochondrial−like	2.127
	A0A3B6CA96	Hsp70−Hsp90 organizing protein−like	2.093
	A0A3B6JIR3	Heat shock cognate 70 kDa protein 2−like	1.571
	A0A3B6KKG9	Heat shock 70 kDa protein, mitochondrial−like	1.547
	F4Y594	Heat shock protein 90	0.348
JM22_F vs. XM33_F	A0A3B6TG72	BOBBER 1−like protein	5.145
	A0A3B5XUY5	DnaJ (HSP40) homolog subfamily B member 4−like	2.125
	A0A3B6GQT8	DnaJ (HSP40) homolog subfamily B member 4−like	1.502
	A0A3B5ZYT4	Heat shock protein 101	0.422
	A0A3B6RDZ6	26.2 kDa heat shock protein, mitochondrial−like	0.190
JM22_C vs. XM33_C	A0A3B6TFV1	26.2 kDa heat shock protein, mitochondrial−like	6.016
	A0A3B6LMB4	SHSP domain−containing protein	1.894
	A0A3B6RHD3	26.2 kDa heat shock protein, mitochondrial−like	1.758
	Q9ZP24	23.6 kDa heat shock protein	1.587
	A0A3B6RDZ6	26.2 kDa heat shock protein, mitochondrial−like	0.235

**Table 4:** Differentially expressed proteins related to cell wall modification in different comparative groups.

Groups	Protein ID	Protein Description	Fold-Change
XM33_F vs. XM33_C	A0A3B6TGN2	Polygalacturonase inhibitor−like	2.222
	A0A3B6H1P4	Germin−like protein	1.955
	A0A1D5V3A3	Pectinesterase inhibitor 7−like	1.852
	A0A3B6TWG3	Xyloglucan endotransglucosylase/hydrolase	1.833
	A0A3B6CBN0	Cinnamyl−alcohol dehydrogenase 7−like	1.506
	A0A3B6TI25	Alpha−1,3−arabinosyltransferase XAT3	0.434
JM22_F vs. JM22_C	A0A3B6TGN2	Polygalacturonase inhibitor−like	1.779
	A0A3B6RK39	Cinnamoyl−CoA reductase 1−like	0.653
	A0A3B6BYY2	Probable cinnamyl−alcohol dehydrogenase 6	0.509
JM22_F vs. XM33_F	A0A3B6TGN2	Polygalacturonase inhibitor−like	1.790
	A0A3B6KU54	Expansin−B4−like	1.657
	A0A3B6IKN1	Endoglucanase	0.653
	A0A3B6TGM9	Probable polygalacturonase isoform X1 protein	0.610
	Q56TP1	Xyloglucan endotransglucosylase/hydrolase	0.605
	A0A3B6TWG3	Xyloglucan endotransglucosylase/hydrolase	0.587
	A0A3B6CBN0	Cinnamyl−alcohol dehydrogenase 7−like	0.556
	A0A3B5Y507	Endoglucanase	0.251
JM22_C vs. XM33_C	A0A3B6B5P1	UDP−arabinopyranose mutase	1.503
	A0A3B6DED8	Xyloglucan endotransglucosylasehydrolase	0.505
	A0A3B6TI25	Alpha−1,3−arabinosyltransferase XAT3−like	0.436
	A0A3B5Y507	Endoglucanase	0.091
	A0A3B6DMR3	Beta−D−xylosidase 4−like	0.052

## Data Availability

The data presented in this study are available on request from the authors.

## Supplementary Materials

The following supporting information can be downloaded at: https://www.mdpi.com/article/10.3390/ijms242115892/s1.
