# Supplementary figures and images for: Label-Free Quantitative Proteomics Reveal the Mechanisms of Young Wheat (Triticum aestivum L.) Ears’ Response to Spring Freezing
Source: Int J Mol Sci. 2023 Nov 2;24(21):15892. doi: 10.3390/ijms242115892 (PMC10648784; doi:10.3390/ijms242115892)

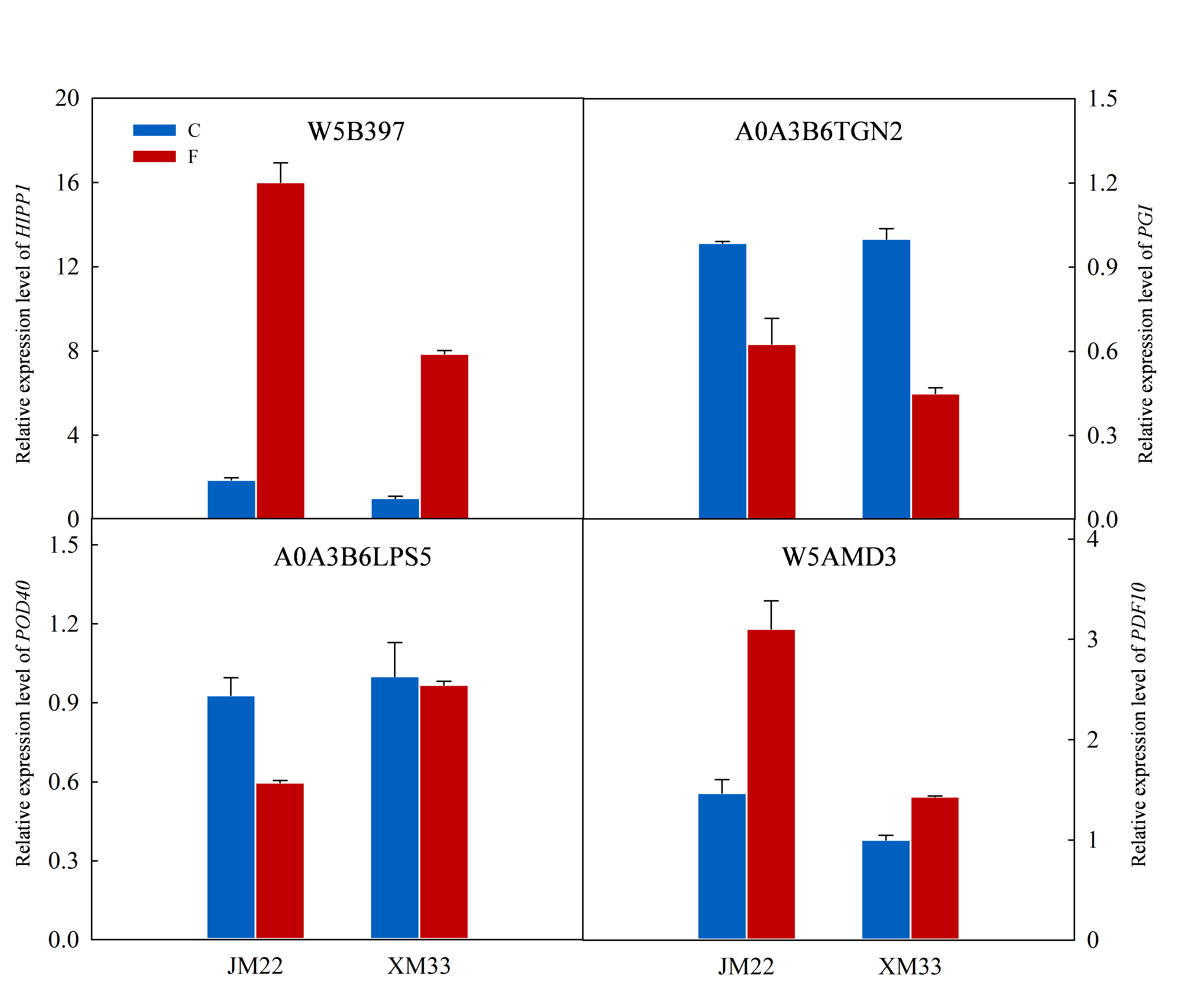

Supplement: Supplementary file 1 [file ijms-24-15892-s001.zip › Figure S2.tif]

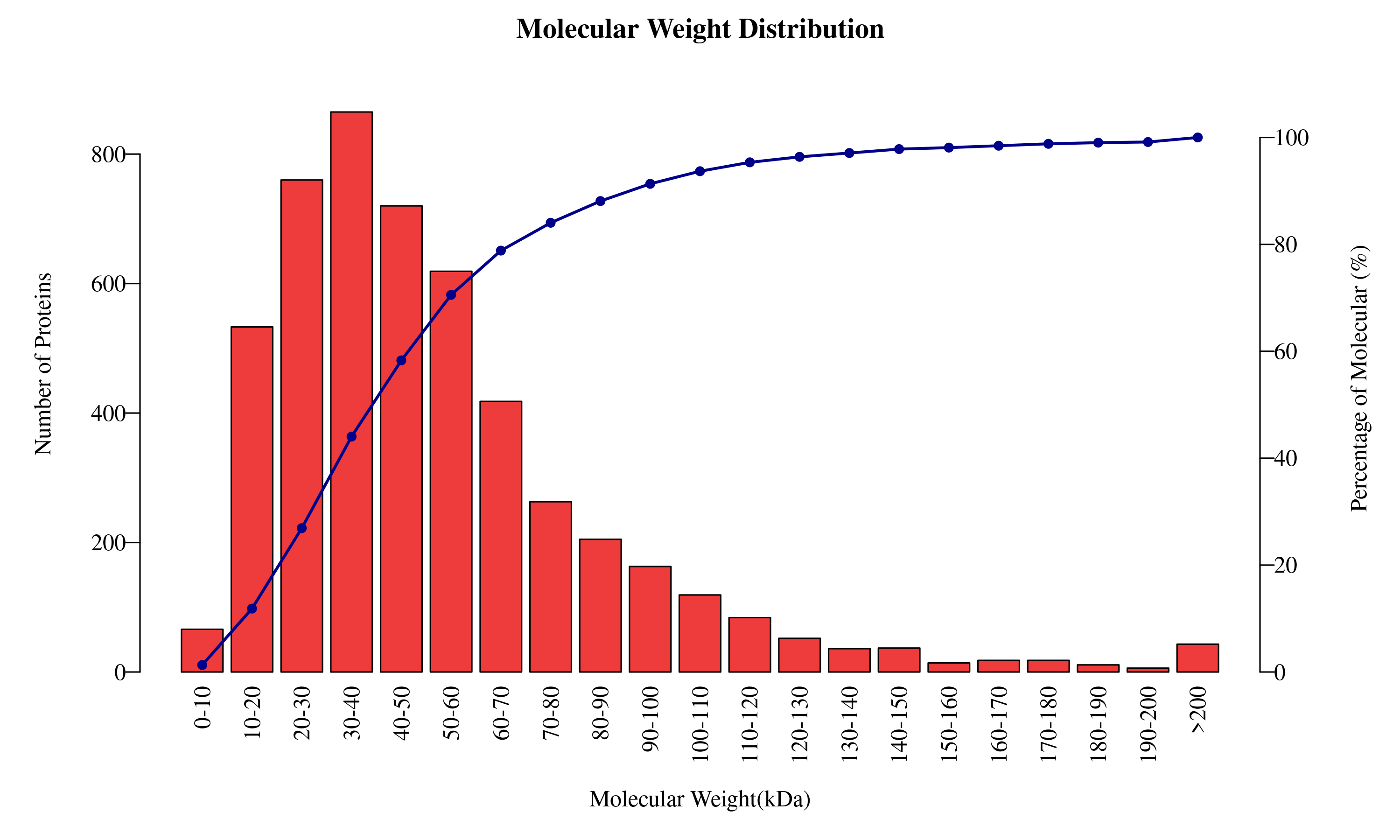

Supplement: Supplementary file 1 [file ijms-24-15892-s001.zip › Figure S1.tif]

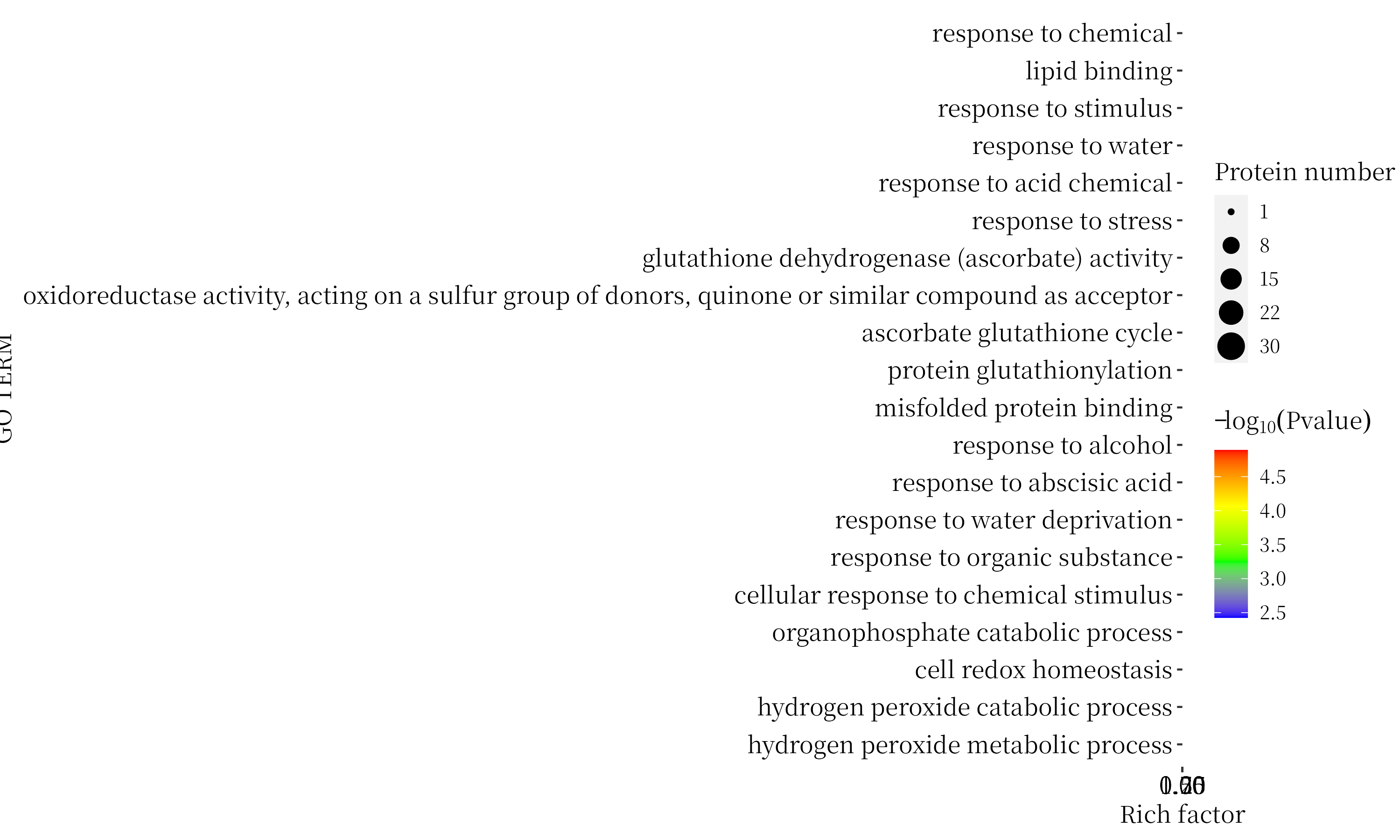

Supplement: Supplementary file 1 [file ijms-24-15892-s001.zip › Figure S3.tif]

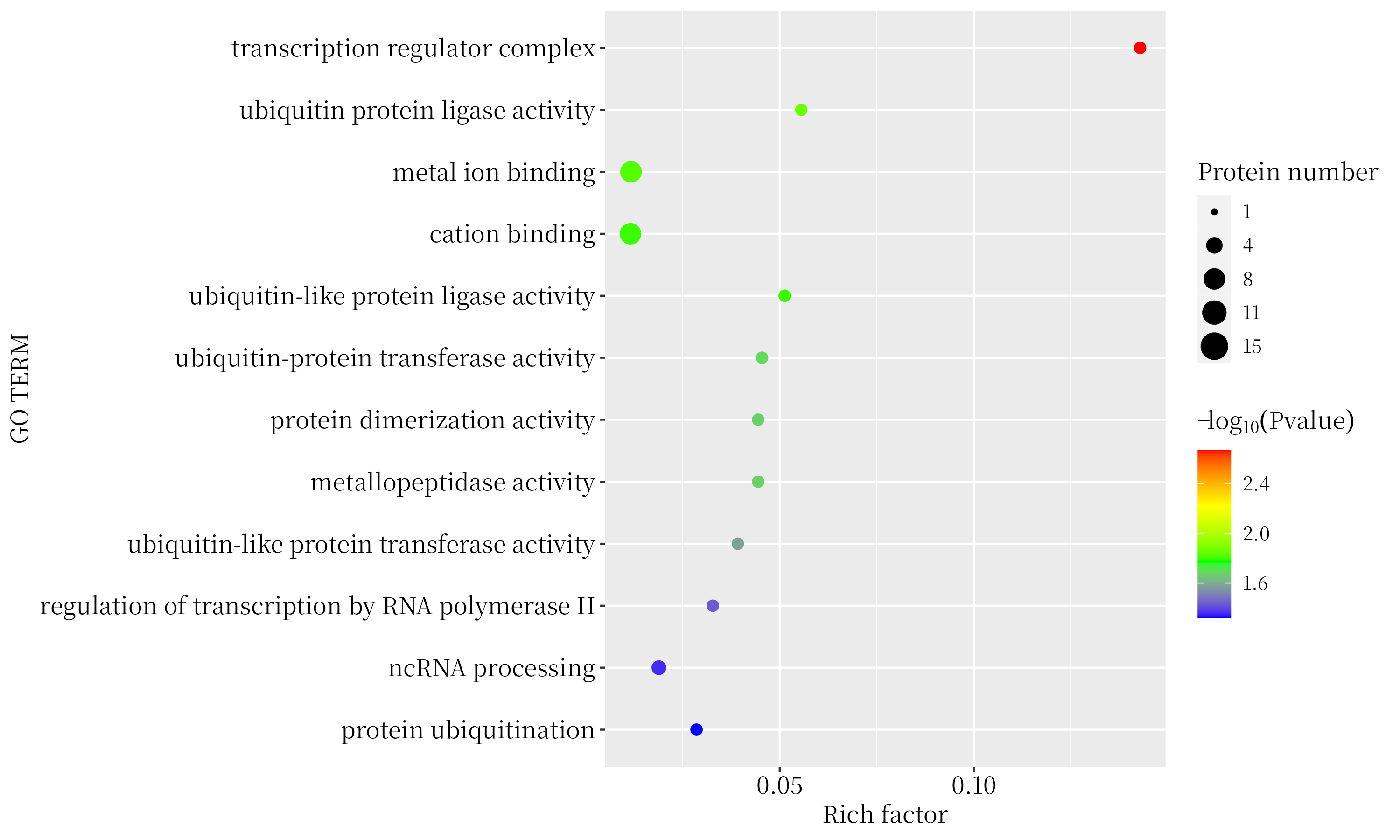

Supplement: Supplementary file 1 [file ijms-24-15892-s001.zip › Figure S4.tif]

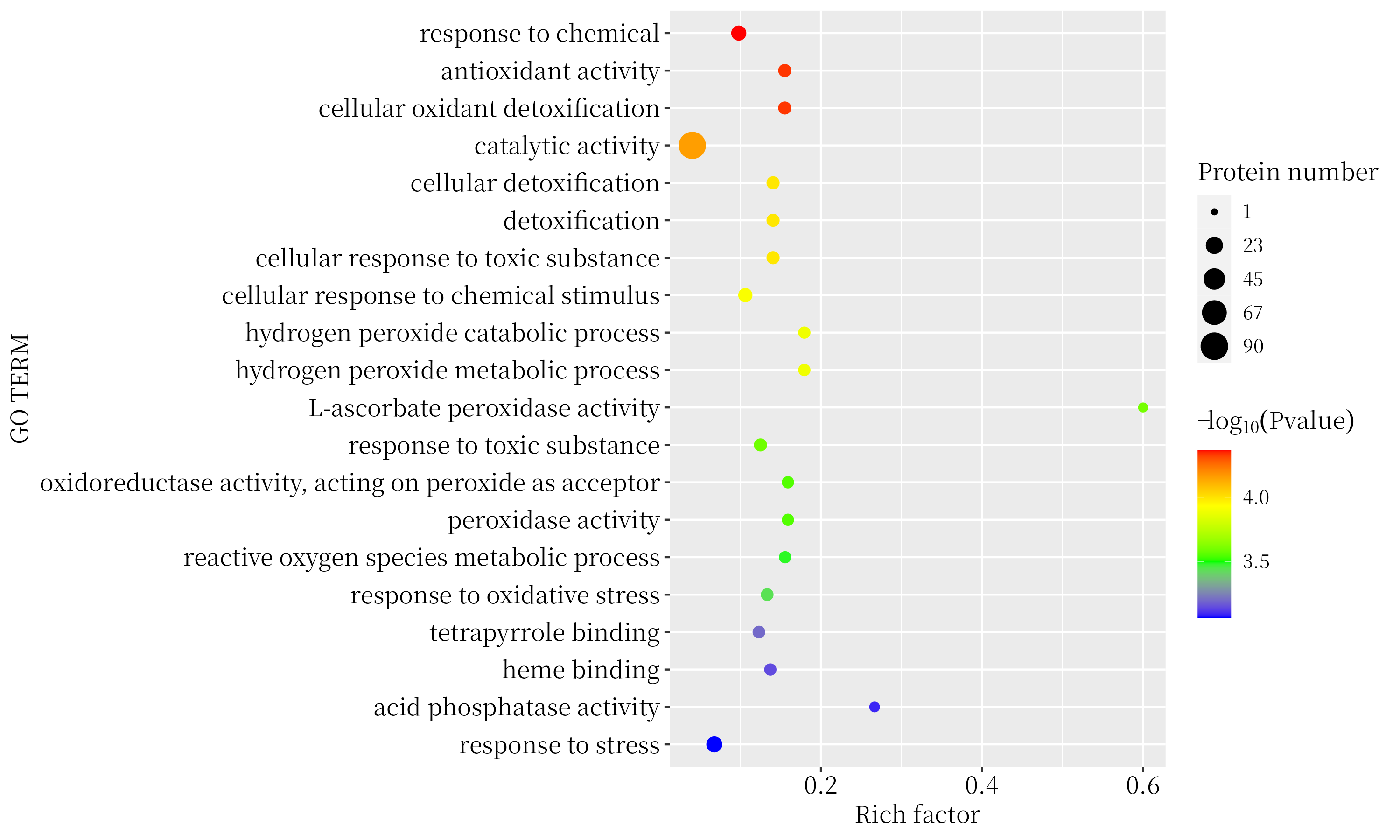

Supplement: Supplementary file 1 [file ijms-24-15892-s001.zip › Figure S5.tif]

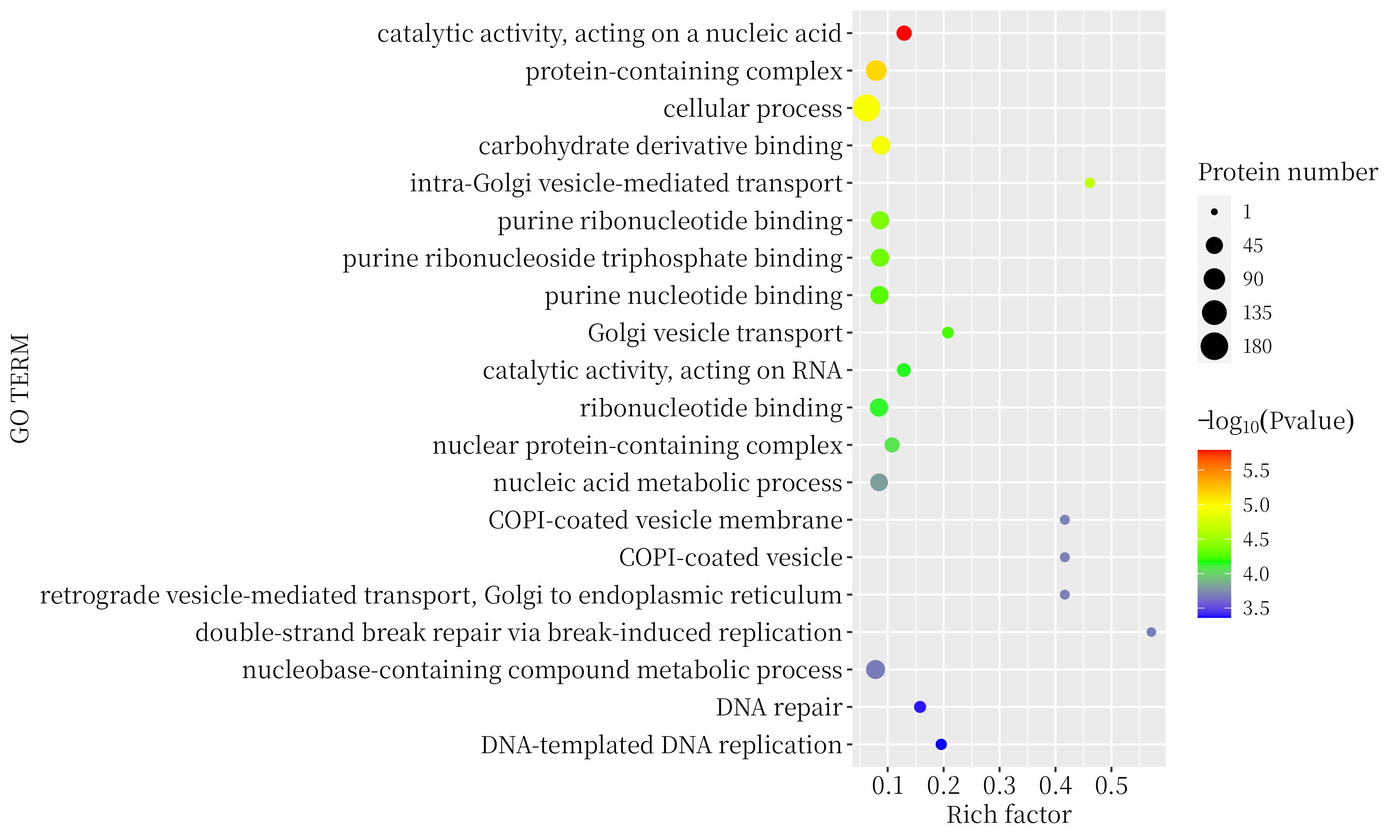

Supplement: Supplementary file 1 [file ijms-24-15892-s001.zip › Figure S6.tif]

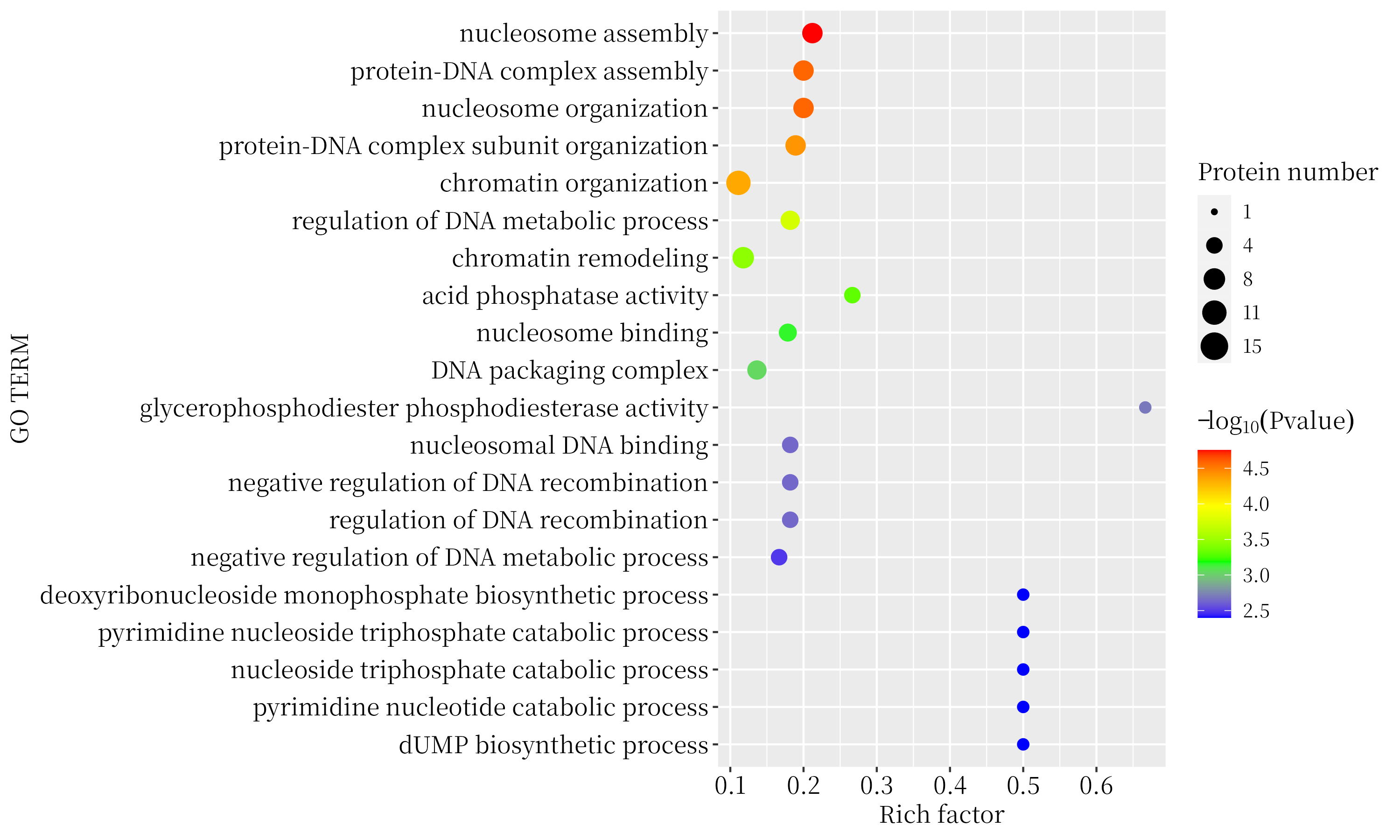

Supplement: Supplementary file 1 [file ijms-24-15892-s001.zip › Figure S7.tif]

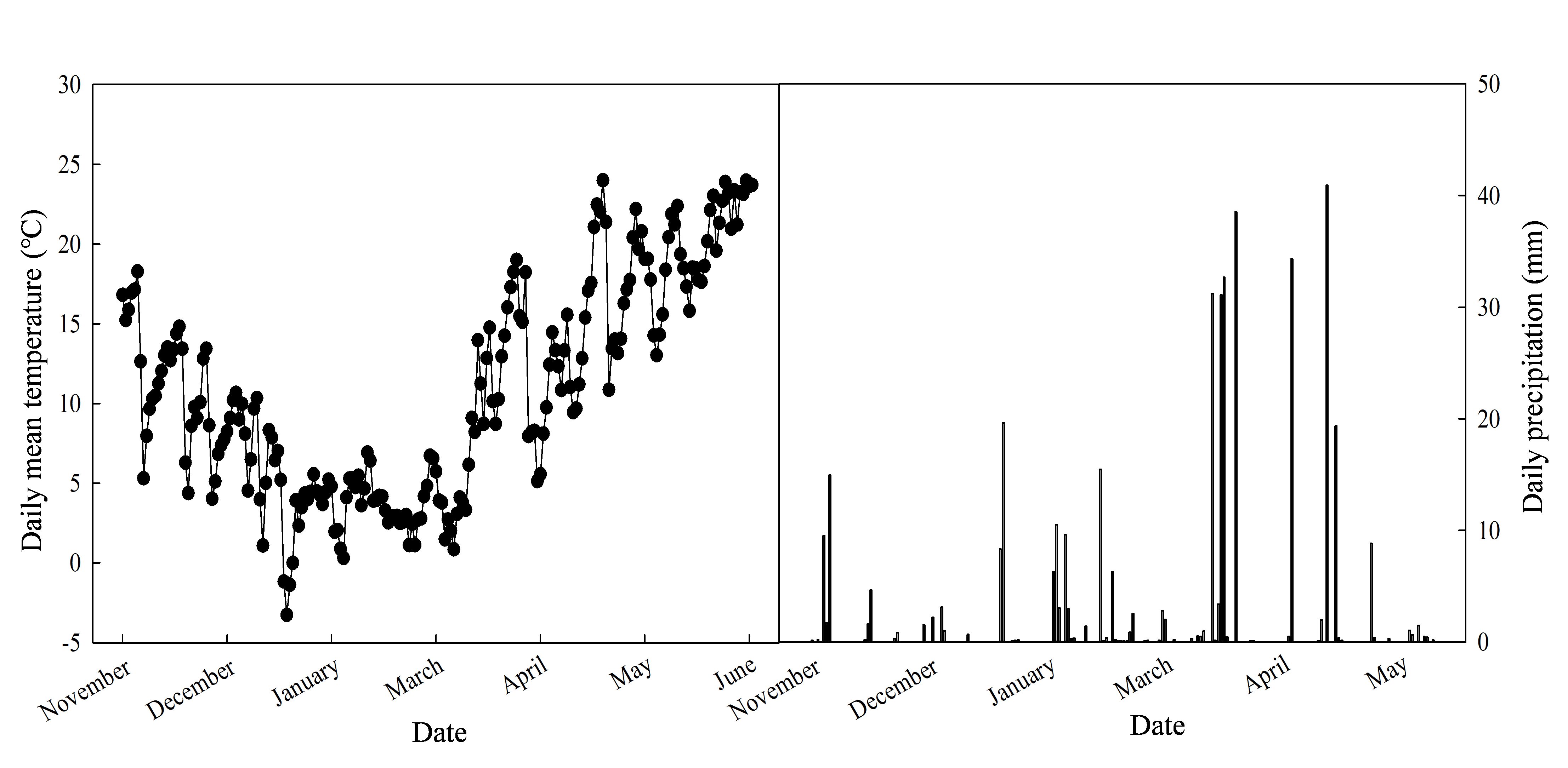

Supplement: Supplementary file 1 [file ijms-24-15892-s001.zip › Figure S8.tif]
